# Lymphadenopathy in Concurrent Head and Neck Malignancies

**DOI:** 10.1155/crra/1544831

**Published:** 2026-01-16

**Authors:** Timothy Fitzgerald, Ryan K. Rigsby

**Affiliations:** ^1^ Department of Radiology, Virginia Commonwealth University, Richmond, Virginia, USA, vcu.edu; ^2^ Department of Radiology and Radiological Sciences, Vanderbilt University Medical Center, Nashville, Tennessee, USA, vanderbilt.edu

**Keywords:** double cancers, lymph node morphology, multiple cancers, second primary malignancy, synchronous malignancies

## Abstract

Second primary malignancies in the head and neck are a major cause of morbidity and mortality and include mucosal epithelial, hematologic, and cutaneous malignancies. Much is known about the imaging features of metastatic cervical lymphadenopathy in a single disease process; however, information on the imaging evaluation of cervical lymph nodes in the setting of multiple concurrent primary cancers is limited. Cancer multiplicity can make imaging evaluation challenging, but accurate interpretation is vital to appropriate workup and treatment. Here, we present four cases of concurrent head and neck malignancies with cervical lymphadenopathy and guidance on how to approach them with attention to lymph node location and morphologic abnormalities.

## 1. Introduction

Second primary malignancies result in significant morbidity and mortality in head and neck cancer patients. The occurrence of second primary malignancies has been attributed to environmental, genetic, and treatment‐related factors. In the head and neck, they can be seen in the setting of upper aerodigestive tract, hematologic, and cutaneous malignancies.

Field cancerization is one explanation for the high rate of primary recurrence and second primary malignancies in head/neck squamous cell carcinoma patients [1]. Long‐term carcinogen exposure to the entire aerodigestive tract, such as from tobacco or alcohol use, results in genetic alterations of multiple “fields” of mucosal epithelial cells. These genetically altered regions can be thought of as “preneoplastic” as they are histologically normal but have a high risk of malignant transformation. The head/neck squamous cell carcinoma 5‐year survival rate of 50% has not significantly improved despite advances in treatment, which is partly attributed to second primary malignancies [2]. The lifetime risk of developing a second primary malignancy in head/neck squamous cell carcinoma patients is 10%–20% and approximately 2%–4% per year [3]. The 20‐year cumulative risk is up to 36% [4].

Hematologic malignancies are also related to second primary malignancies. Patients with non‐Hodgkin lymphoma have a significantly increased risk of developing a second primary malignancy in the lip, tongue, oropharynx, skin, and thyroid, as well as secondary lymphomas [5, 6]. Improved lymphoma treatments have led to improved survival, but the increased lifespan comes with an increased risk of second primary malignancy development [6].

Cutaneous neoplasms can also present as second primary malignancies or be a risk factor for second primary malignancy development. Patients with multiple basal cell carcinomas have an increased risk of additional cutaneous cancers as well as colon and hematologic malignancies [7]. Multiple cutaneous squamous cell carcinomas portend not only a higher likelihood of local recurrence and nodal metastasis but also an increased risk of subsequent cutaneous squamous cell carcinoma and melanoma formation [8]. Likewise, patients with melanoma are at increased risk of developing a second primary melanoma [9] and cutaneous squamous cell carcinoma [10].

A second primary malignancy can be challenging to recognize on imaging, but its identification is vital. When a second primary malignancy is suspected and lymphadenopathy is present, it is worthwhile to attempt to determine which primary tumor has metastasized to which abnormal lymph nodes, as this determines which node or nodes should be targeted for fine needle aspiration or core needle biopsy to confirm the diagnosis. This in turn provides valuable information for treatment planning. While much is known about the imaging features of lymph nodes in single disease processes, less is known about the imaging of lymph nodes in the setting of multiple concurrent malignancies. Here, we present four cases of patients with concurrent primary malignancies of the head and neck with lymphadenopathy and guidance on how to approach them. Due to the retrospective nature of this study with the collection of only deidentified information, an Institutional Review Board waiver was granted, and therefore, written informed consent was not obtained.

## 2. Case 1: Enlarged, Rounded Nodes in Unexpected Locations for Metastases

**Figure 1:**
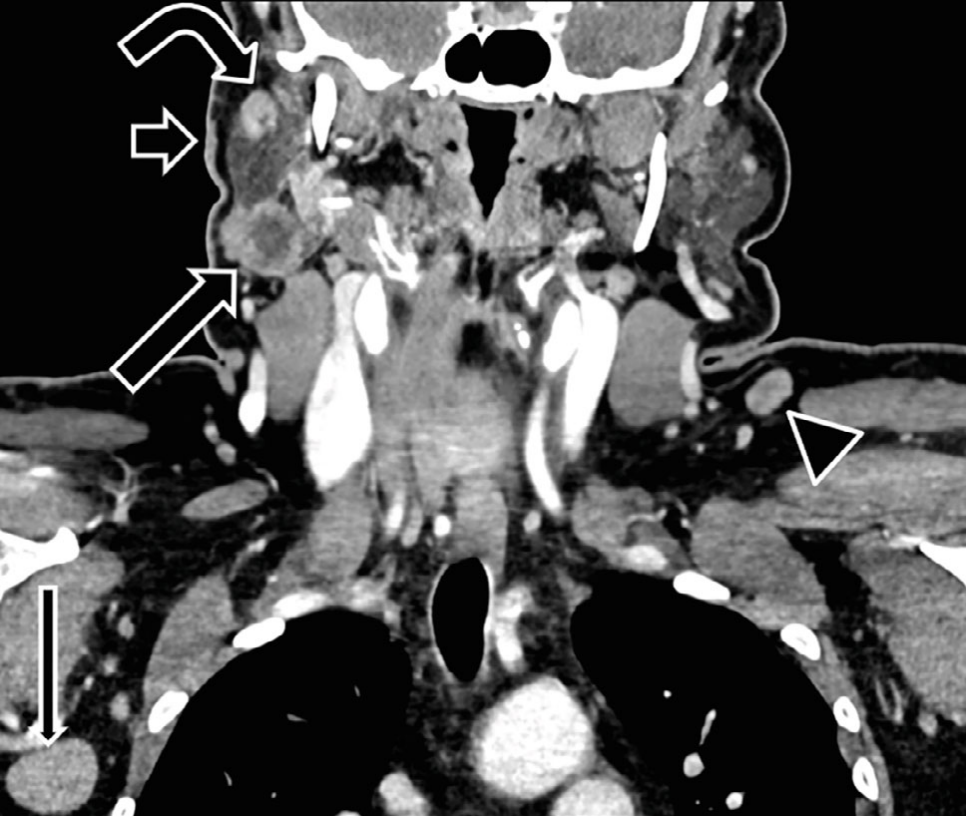
A 66‐year‐old female with a history of basal cell carcinoma presented with a right parotid region lump. Coronal contrast‐enhanced soft tissue neck CT shows a centrally necrotic right parotid tail lesion (long arrow). An enlarged superficial parotid lymph node is present superiorly (curved arrow). Careful inspection reveals skin thickening overlying the parotid, which was the site of the patient′s prior basal cell carcinoma resection (short arrow). Enlarged, rounded nonnecrotic nodes are present in left Level V (arrowhead) and the right axilla (thin long arrow), which is not a typical pattern for disease spread from a right preauricular skin primary. Right parotidectomy showed metastatic basal cell carcinoma and follicular lymphoma, and nodes from right neck dissection showed follicular lymphoma. Notably, the lymphoma was a new diagnosis for this patient.

A 66‐year‐old female with a previously resected right preauricular cutaneous basal cell carcinoma presented to the otolaryngology clinic with a slowly enlarging “lump” behind the right mandibular angle. Contrast‐enhanced computed tomography of the neck soft tissues was performed (Figure 1) and demonstrated a 2.7 cm centrally necrotic mass in the right parotid tail. More superiorly within the parotid gland, there was a mildly enlarged lymph node measuring up to 1.3 cm. Subtle preauricular skin thickening was noted at the prior basal cell carcinoma resection site. A mildly enlarged right Level IIA lymph node measuring up to 1.7 cm in maximum dimension was present. Additionally, bilateral enlarged, rounded nodes were noted in Level VA and in the right axilla.

The patient underwent right parotidectomy and neck dissection. The parotid mass and three additional parotid lymph nodes demonstrated metastatic basal cell carcinoma on pathology. These nodes also harbored Grade 1–2 follicular lymphoma. Right Level II–IV nodes were negative for basal cell carcinoma metastasis but were diffusely involved by follicular lymphoma. Lymphoma was a new diagnosis for this patient. It was thought that the patient′s lymphoma‐induced immune dysregulation contributed to the advanced basal cell carcinoma [11, 12].

## 3. Case 2: Focal Nodal Hyperenhancement on a Background of Diffusely Enlarged, Homogeneous, Rounded Nodes

**Figure 2:**
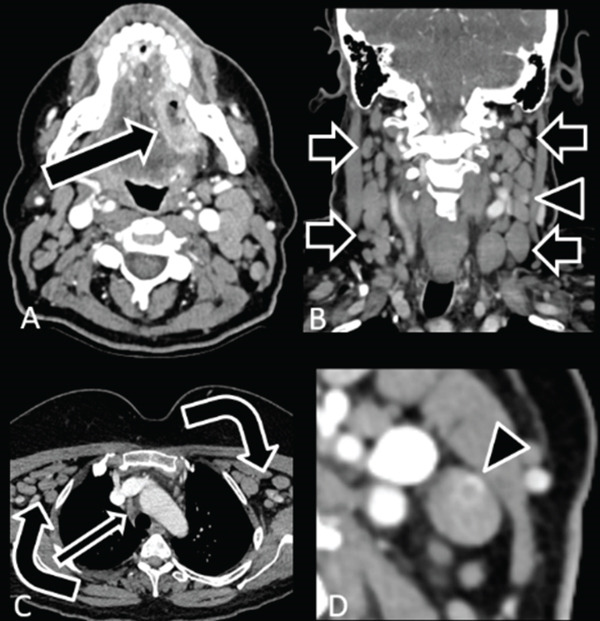
A 53‐year‐old female with a history of chronic lymphocytic leukemia (CLL) presented with biopsy‐proven left oral tongue squamous cell carcinoma (SCC). (A) Axial contrast‐enhanced soft tissue neck CT shows an enhancing ulcerated mass in the left oral tongue (arrow). (B) Coronal image demonstrates diffuse bilateral nonnecrotic lymphadenopathy in the neck (short arrows), which is typical for leukemia/lymphoma and not typical for metastatic SCC. (C) Axial image at the level of the upper chest shows nonnecrotic bilateral axillary lymphadenopathy (curved arrows) and mediastinal lymphadenopathy (thin arrow), typical for leukemia/lymphoma. In this case, node size cannot be reliably used to assess for metastatic SCC. Careful inspection, however, reveals a small internal ring of enhancement in a left Level IIB node: (B) arrowhead in coronal image and (D) arrowhead in axial image, which is less typical for leukemia/lymphoma.

A 53‐year‐old female with a history of chronic lymphocytic leukemia presented to the otolaryngology clinic with biopsy‐proven left oral tongue squamous cell carcinoma. Contrast‐enhanced computed tomography (Figure 2) demonstrated an enhancing ulcerated mass in the left oral tongue. Enlarged nonnecrotic lymph nodes were present throughout the neck, some with a rounded morphology, compatible with known leukemia. The lymph nodes were internally homogeneous, except for a single left Level IIB node, which demonstrated a 0.4 cm internal ring of hyperenhancement.

Subsequent left oral cavity composite resection and left neck dissection were performed. Pathology confirmed squamous cell carcinoma in the oral tongue and chronic lymphocytic leukemia in the neck nodes. A single lymph node of 63 was positive for both chronic lymphocytic leukemia and metastatic squamous cell carcinoma.

## 4. Case 3: Nodal Calcifications and a Necrotic Node in an Unexpected Location for Metastasis

**Figure 3:**
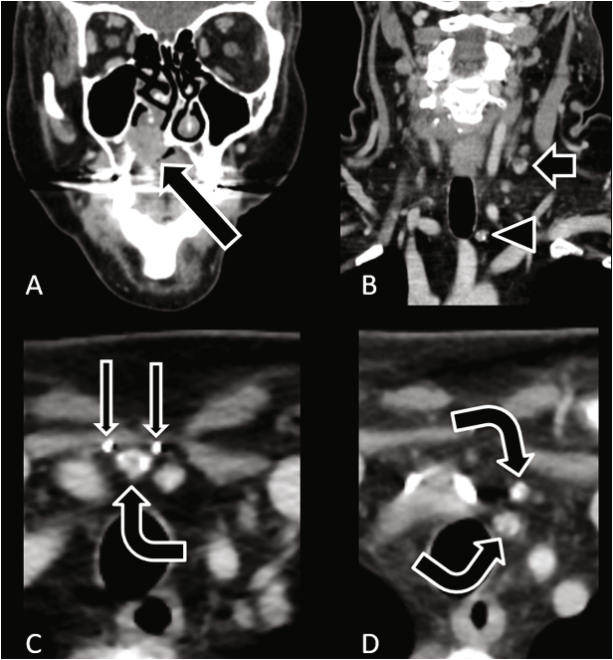
A 60‐year‐old female with a history of papillary thyroid carcinoma (PTC) in remission after treatment 20 years prior presented with biopsy‐proven adenoid cystic carcinoma (ACC) of the palate. (A) Coronal contrast‐enhanced soft tissue neck CT shows an aggressive enhancing soft tissue mass centered on the right hard palate with erosion into the nasal cavity (arrow). (B) Coronal image of the neck shows a necrotic left Level III node (short arrow), which would be atypical for metastatic disease from a right palate mass, and a Level VI node with internal calcifications (arrowhead). (C, D) Axial images of the lower neck reveal an absent thyroid with surgical clips (thin arrows) and multiple calcified lymph nodes (curved arrows). Nodal calcifications are most common in metastatic thyroid carcinomas. Palate mass resection showed ACC, and neck dissection showed metastatic PTC in multiple nodes, including in Level VI and left Level III.

A 60‐year‐old female with a history of thyroid cancer treated 20 years prior presented to the otolaryngology clinic for evaluation of biopsy‐proven adenoid cystic carcinoma of the palate. Contrast‐enhanced computed tomography (Figure 3) showed an enhancing soft tissue mass in the right hard palate eroding into the nasal cavity. There was a 1.2 cm long‐axis necrotic left Level III lymph node, multiple Level VI lymph nodes with internal calcifications, and a surgically absent thyroid.

Partial maxillectomy and bilateral neck dissections confirmed adenoid cystic carcinoma of the palate. Metastatic papillary thyroid carcinoma was identified in four nodes, including in Level VI and left Level III.

## 5. Case 4: Abnormal Node Location in the Setting of Multiple Cutaneous Malignancies and the Limits of Imaging Extranodal Extension

**Figure 4:**
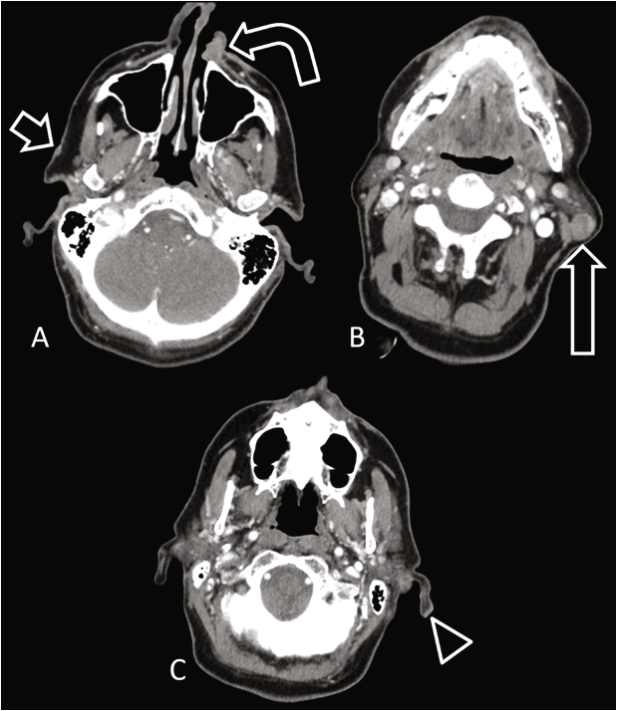
A 74‐year‐old male with a history of left nasal ala cutaneous squamous cell carcinoma (SCC), right preauricular cutaneous SCC, and left pinna melanoma presented with a palpable left upper neck mass. (A) Axial contrast‐enhanced neck soft tissue CT shows left nasal ala destruction with an enhancing soft tissue mass (curved arrow) and a small right preauricular skin lesion (short arrow). (B) An enlarged, rounded lymph node in the left parotid tail is noted. This would be an atypical site for metastatic disease from either SCC primaries. (C) Careful inspection shows subtle right inferior helix skin thickening (arrowhead), which would be easy to overlook if the history of melanoma was unknown. Parotid node fine needle aspiration followed by partial parotidectomy confirmed metastatic melanoma.

A 74‐year‐old male with multiple cutaneous cancers, including a left nasal ala squamous cell carcinoma, right preauricular squamous cell carcinoma, and left pinna melanoma, as well as a palpable abnormality in the left upper neck, presented to the surgical oncology clinic. Contrast‐enhanced computed tomography (Figure 4) demonstrated a small right preauricular skin lesion, a destructive left nasal ala soft tissue mass, a rounded, enlarged left parotid tail lymph node, and a subtle skin thickening of the left inferior helix.

Patient underwent left parotid node fine needle aspiration followed by right preauricular squamous cell carcinoma wide excision, partial rhinectomy, left pinna melanoma wide excision, partial left parotidectomy, and left neck dissection. The left parotid node contained metastatic melanoma with extranodal extension.

## 6. Discussion

Concurrent malignancies involving the head and neck are uncommon but not rare. Clinical history is vital to recognizing that two malignancies may be present, though imaging may be the first indication of a second primary malignancy, such as in Case 1. Lymphadenopathy in this setting can be challenging to evaluate on imaging. However, knowledge of lymph node drainage pathways and abnormalities of node size, shape, cystic/necrotic change, hyperenhancement, calcification, and extranodal extension are important to generate an accurate report that guides the next step in management, particularly determining which node or nodes to target for percutaneous sampling.

### 6.1. Drainage Pathways

Knowledge of lymph node drainage pathways is crucial for recognizing when an abnormal lymph node is in an unexpected location for metastasis for a given primary malignancy. In Case 1, metastatic disease to left Level V and right axillary nodes from a right preauricular skin primary malignancy would be unusual. In Case 3, a right palate primary malignancy would be unlikely to metastasize only to Level VI and left Level III. In Case 4, while the pinna drains to parotid lymph nodes, the left side of the nose, where there was concurrent squamous cell carcinoma, drains to left Level IB then II [13]. Lymphatic drainage pathways have been well described and are summarized in Table 1.

**Table 1:** Lymph node levels and drainage pathways [13–16].

**Level**	**Anatomic location**	**Drainage pathways**
IA (submental) IB (submandibular)	Below the mylohyoid muscle • IA: Between the anterior bellies of the digastric muscles • IB: Lateral to anterior belly of digastric muscle, anterior to submandibular gland posterior margin	IA drainage from: Middle lower lip, mentum, anterior FOM, tongue tip, sublingual glands IB drainage from: IA, mandibular and buccinator nodes; eyelids∗, conjunctiva∗, anterior cheek∗, nose∗, upper lip, lateral lower lip, anterior nasal cavity, lower gingiva, anterior 2/3 tongue minus tip, posterior FOM, submandibular glands Drains to: IA to IB, IB to II
II (upper jugular)	Between submandibular gland posterior margin and SCM posterior margin, superior to hyoid bone inferior margin • IIA: Abutting or anterior to IJV posterior margin • IIB: Posterior to IJV posterior margin	Drainage from: IB, parotid∗, preauricular, mastoid∗, occipital, retropharyngeal, and superficial cervical nodes; deep masticator space, upper gingiva, hard palate, pharynx∗, posterior 1/3 tongue, supraglottis Drains to: III
III (middle jugular)	Between the anterior and posterior margins of SCM, between the hyoid bone inferior margin and the cricoid cartilage inferior margin	Drainage from: II; glottis, subglottis∗, thyroid∗, cervical esophagus∗ Drains to: IV
IV (lower jugular)	Between the cricoid cartilage inferior margin and clavicle, between the CCA medial margin and a line from the posterolateral SCM to the lateral anterior scalene muscle (includes medial supraclavicular nodes)	Drainage from: III, V, VI∗; chest, abdomen, pelvis Drains to: Thoracic duct, right lymphatic duct
VA/B (spinal accessory)	Anterior to trapezius muscle • VA: Between the cricoid cartilage inferior margin and skull base, posterior to the SCM posterior margin • VB: Between cricoid cartilage inferior margin and clavicle, posterior/lateral to a line from posterolateral SCM to lateral anterior scalene muscle (includes lateral supraclavicular nodes)	Drainage from: Parotid nodes∗, occipital nodes∗; pharynx∗, regional skin Drains to: IV
VI (visceral)	Between the hyoid bone inferior margin and the manubrium superior margin, medial to the CCA medial margins (includes the Delphian node)	Drainage from: Thyroid∗, cervical esophagus∗, subglottis∗, trachea∗ Drains to: IV, superior mediastinal
Parotid	Within or adjacent to the parotid gland, multiple groups • Superficial extrafascial (includes preauricular) • Subfascial extraglandular • Deep intraglandular	Drainage from: Mastoid∗ and malar nodes; frontal scalp∗, parietal scalp∗, temporal scalp∗, anterior pinna, anterior EAC∗, eyelids∗, conjunctiva∗, posterior cheek∗, soft palate∗, orbit∗, middle ear cavity, eustachian tubes∗ Drains to: II, superficial cervical
Mastoid (posterior auricular, retroauricular)	Superficial to the temporal bone mastoid process	Drainage from: Parietal scalp∗, temporal scalp∗, posterior pinna, posterior EAC Drains to: II, parotid
Occipital	In the soft tissues overlying the posteroinferior calvarium (occipital and suboccipital)	Drainage from: Occipital and suboccipital regions Drains to: II
Retropharyngeal	Retropharyngeal space (includes medial and lateral nodes; lateral includes node of Rouvière)	Drainage from: Posterior 2/3 nasal cavity∗, paranasal sinuses∗, soft palate∗, pharynx∗, eustachian tubes∗, thyroid∗ Drains to: II
Facial	Multiple groups named for adjacent structures, nodes are superficial • Mandibular • Buccinator • Infraorbital (nasolabial) • Malar (includes zygomatic)	Drainage from: Eyelids∗, conjunctiva∗, nose∗, cheek∗ Drains to: • Mandibular drains to: IB • Buccinator drains to: IB • Infraorbital drains to: Buccinator and mandibular • Malar drains to: Parotid
Superior mediastinal (VII)	Below the manubrium superior margin	Drainage from: VI∗; thyroid∗, cervical esophagus∗, trachea∗ Drains to: Thoracic duct, right lymphatic duct
Superficial cervical	Superficial to SCM	Drainage from: Parotid∗, lower pinna Drains to: II

*Note:* “Pharynx” refers to the nasopharynx, oropharynx, and hypopharynx. An individual structure may drain directly to multiple lymph node groups, and the drainage pathways in this table have been simplified. For example, lymphatics of the sublingual glands drain directly to both Levels IA and IB; however, for simplicity and because Level IA typically drains to Level IB, the submandibular glands are not listed under Level IB. The asterisk indicates that the structure is listed more than once for “drainage from.”

Abbreviations: CCA, common carotid artery; EAC, external auditory canal; FOM, floor of mouth; IJV, internal jugular vein; SCM, sternocleidomastoid muscle.

### 6.2. Size

Size is arguably the least helpful feature of an abnormal lymph node. Cervical lymph nodes have classically been evaluated in the long axis with enlargement considered > 15 mm for Levels I and II, > 8 mm for retropharyngeal nodes, and > 10 mm elsewhere [15]. Many different size criteria have been proposed based on nodal site, age, and plane of measurement; however, the overall error rates are high regardless of the criteria applied with false‐positive and false‐negative rates of 15%–20% [13]. It has been reported that in head/neck squamous cell carcinoma patients, 50% of metastatic nodes measure < 5 mm [17]. In Case 2, size could not be reliably used as an indicator of metastatic squamous cell carcinoma due to diffuse nodal enlargement from leukemia. In Case 3, all of the calcified Level VI lymph nodes measured no more than 8 mm in long axis but contained metastatic papillary thyroid carcinoma.

### 6.3. Shape

Normal lymph nodes have a reniform shape with a fatty hilum. Reactive lymph nodes are increased in size but tend to maintain a normal shape [15]. If normal nodal tissue is infiltrated by tumor, however, the fatty hilum tends to be replaced, and the node capsule eccentrically expands, resulting in a rounded shape [14]. Case 1 showed rounded and enlarged nodes in the left Level V and the right axilla. Case 2 showed multiple bilateral, rounded, and enlarged nodes. Case 4 demonstrated a rounded left parotid node.

### 6.4. Cystic/Necrotic Change

Nodal necrosis is identified by central nonenhancement or hypoenhancement with a variably thick and enhancing surrounding nodal cortex. When central nodal necrosis is > 3 mm, it is routinely identified on contrast‐enhanced computed tomography and is the most reliable imaging finding of metastatic disease in the setting of head/neck squamous cell carcinoma regardless of lymph node size [13] with a 95%–100% specificity for metastatic disease [14]. Case 3 showed a necrotic left Level III node. In small lymph nodes, however, normal hilum volume averaging can mimic necrosis. Cystic nodal disease is characterized by central fluid attenuation with thin peripheral enhancement. In adult patients, a new cystic lesion should raise concern for metastatic disease, particularly human papillomavirus–positive oropharyngeal squamous cell carcinoma or papillary thyroid carcinoma [15], which can present with purely cystic nodal metastasis [14]. There is a differential diagnosis for cystic/necrotic lesions of the neck, including other neoplasms, suppurative lymphadenitis, and branchial cleft cysts.

### 6.5. Hyperenhancement

The most common cause of nodal hyperenhancement is acute infection, which presents with a homogeneous reactive node. Metastatic papillary and medullary thyroid carcinomas, head/neck squamous cell carcinoma, renal cell carcinoma, Kaposi sarcoma, and lymphoma can also cause nodal hyperenhancement [13]. In Case 2, the focal internal enhancement in a left Level IIB node, which was not seen in other leukemic nodes, suggested metastatic squamous cell carcinoma. Hypervascularity can also be seen in the setting of recent chemotherapy or radiation therapy.

### 6.6. Calcification

Cervical nodal calcifications are uncommon and usually reflect metastatic papillary or medullary thyroid carcinoma [14]. Tuberculosis and previously treated infections can also cause nodal calcifications. While remote granulomatous disease can cause calcifications, this is much less common in the neck than in the chest [14], so such neck nodes should not be quickly dismissed as benign. Nodal calcifications can also occur in treated lymphoma, metastatic mucinous adenocarcinomas from outside the head and neck, and less commonly in head/neck squamous cell carcinoma [13]. In Case 3, the radiologist recognized that the calcified Level VI nodes were concerning for recurrent thyroid carcinoma.

### 6.7. Extranodal Extension

As a metastatic nodal deposit grows, it can extend beyond the margin of the capsule, known as extranodal extension. On contrast‐enhanced computed tomography, extranodal extension is suggested by a thickened nodal capsule with irregular or ill‐defined margins, stranding in the adjacent fat, frank invasion of an adjacent structure, or a matted appearance where three or more nodes abut one another. The specificity of extranodal extension on imaging is high for human papillomavirus–negative oral cavity and larynx squamous cell carcinoma at about 93% in one meta‐analysis [18]. However, the specificity for human papillomavirus–positive oropharyngeal squamous cell carcinoma is only 72%, so caution should be used when suggesting extranodal extension on imaging in this setting. Approximately 45% of pathology‐proven nodes with extranodal extension do not have a computed tomography correlate [17]. This was true in Case 4, where the left parotid node was well marginated on computed tomography but showed extranodal extension on histology. About half of the nodes measuring 2–3 cm and about three‐quarters of the nodes > 3 cm demonstrate extranodal extension [13]. Notably, 25% of nodes with extranodal extension measure < 10 mm [17].

Table 2 reviews patterns of lymphadenopathy in the neck, summarizes the current cases, and references additional cases in the literature.

**Table 2:** General patterns of cervical lymphadenopathy, summary of cases, and additional cases in the literature.

**Pathology**	**Lymph node characteristics [** **13** **–** **15** **]**	**Typical location [** **13** **–** **15** **]**	**Case number and diagnosis timeline** ^**a**^	**Additional cases of simultaneous cancers in the head/neck with node involvement and with imaging in the literature** ^**b**^
SCC in the head/neck	Enlarged, rounded, necrotic, can be purely cystic	Usually ipsilateral to the primary malignancy in an expected drainage pathway (if bilateral, then metastatic burden tends to be greater ipsilateral to the primary malignancy)	2: SCC was suspected clinically and confirmed on biopsy prior to imaging	SCC and thyroid CA [19–21] SCC, thyroid CA, and lymphoma [22] SCC and lymphoma [23–28] Multiple SCCs [29–41]
Thyroid CA	Calcifications, enlarged, rounded, can be necrotic, can be purely cystic	Levels III, IV, and VI, superior mediastinal and retropharyngeal, commonly bilateral	3: Metastatic papillary thyroid CA was suspected by the radiologist on preoperative imaging and pathologically confirmed after neck dissection	SCC and thyroid CA [19–21] SCC, thyroid CA, and lymphoma [22] Thyroid CA and lymphoma [42–46] Multifocal thyroid CA [47–51] Thyroid CA and parathyroid CA [52, 53]
Lymphoma or leukemia	Enlarged, rounded, typically internally homogeneous	Bilateral and diffuse	1: Lymphoma diagnosis was unknown prior to neck dissection 2: Leukemia diagnosis was known prior to neck dissection	SCC, thyroid CA, and lymphoma [22] SCC and lymphoma [23–28] Thyroid CA and lymphoma [42–46]
Reactive	Enlarged, normal reniform shape, internally homogeneous, can hyperenhance	Bilateral or unilateral	N/A	N/A

Abbreviations: CA, carcinoma; N/A, not applicable; SCC, squamous cell carcinoma.

^a^For Case 4, metastatic melanoma was clinically suspected and confirmed on fine needle aspiration prior to surgery. The multiple cutaneous SCCs were clinically suspected, and biopsy confirmed prior to surgery.

^b^Additionally, there are cases of multiple concurrent parotid carcinomas in the literature [54, 55].

### 6.8. Limitations of Imaging

There are limitations in the imaging evaluation of abnormal lymph nodes. Lymphatic drainage pathways are altered in the postsurgical neck, so recurrent malignancy may present in an unexpected location. Nodal hyperenhancement and calcifications are nonspecific findings with differential diagnoses. Size and extranodal extension have significant limitations, as discussed above. Additionally, while F‐18 fluorodeoxyglucose positron emission tomography improves the sensitivity and specificity of nodal metastasis detection compared to computed tomography, magnetic resonance imaging, and ultrasound, it fails to detect nodal metastasis in half of patients with a clinically node‐negative neck [56].

## 7. Conclusion

Concurrent malignancies of the head and neck are uncommon but not rare. Lymphadenopathy in this setting can be challenging to evaluate. Knowledge of normal lymph node drainage patterns and specific nodal morphologic abnormalities is important to detect the presence of multiple malignancies and to generate an actionable report.

## Conflicts of Interest

The authors declare no conflicts of interest.

## Funding

No funding was received for this manuscript.

## Data Availability

The data are not publicly available due to privacy or ethical restrictions.
